# Supplementary material for: Immunogenic amino acid motifs and linear epitopes of COVID-19 mRNA vaccines
Source: PLoS One. 2021 Sep 9;16(9):e0252849. doi: 10.1371/journal.pone.0252849 (PMC8428655; doi:10.1371/journal.pone.0252849)
Supplement: S1 Fig — (PDF) [file pone.0252849.s001.pdf]

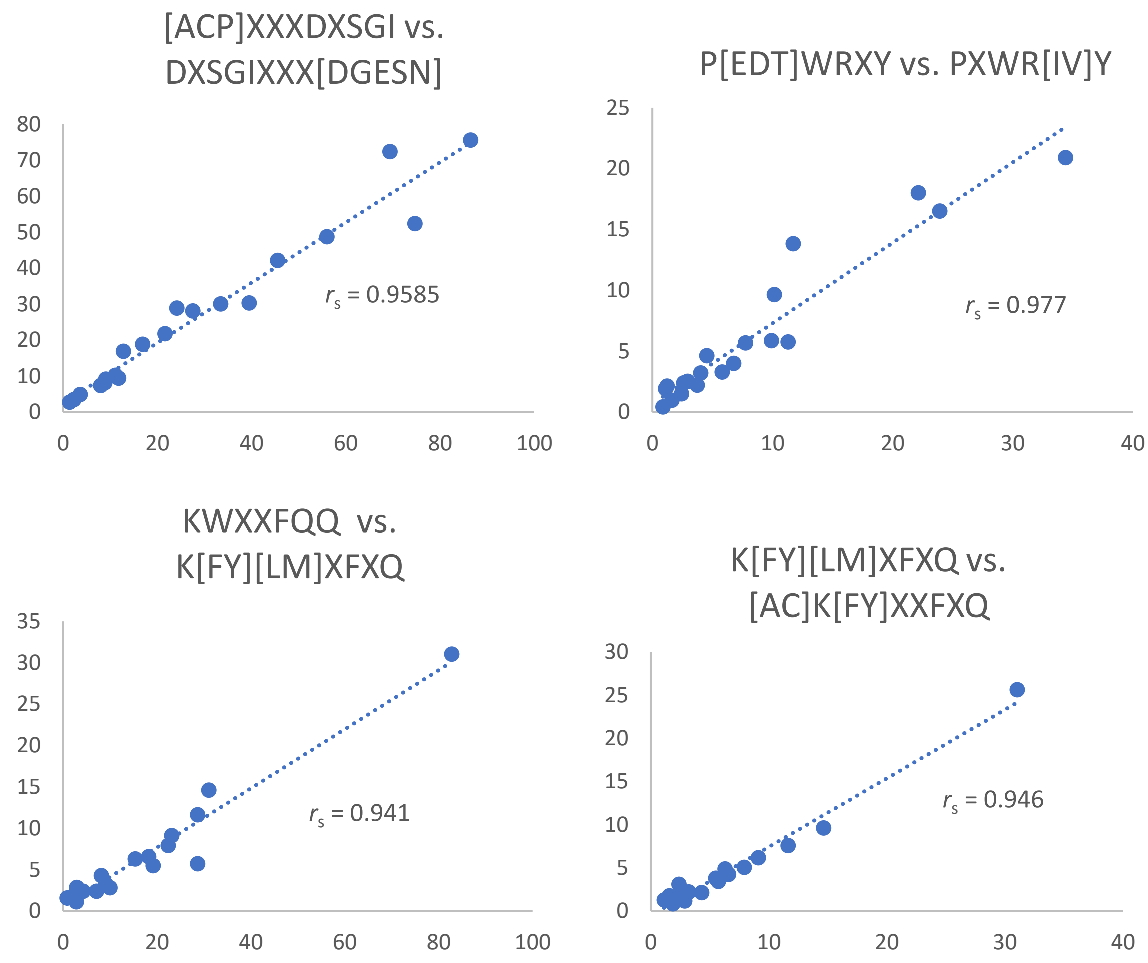

S1 Fig. Correlation of IgG recognition of peptide motifs with overlapping amino acids. The fold enrichment in IgG with specificity for the different pairs of amino acid motifs (respective x and Y-axis) are shown along with the Spearman rank correlation (r_s) value. Each symbol represents a different COVID-19 mRNA vaccine recipient. Trend lines were fitted by linear regression. Spearman rank correlation (r_s) is displayed.
