# Supplementary material for: Immunogenic amino acid motifs and linear epitopes of COVID-19 mRNA vaccines
Source: PLoS One. 2021 Sep 9;16(9):e0252849. doi: 10.1371/journal.pone.0252849 (PMC8428655; doi:10.1371/journal.pone.0252849)
Supplement: S2 Fig — (PDF) [file pone.0252849.s002.pdf]

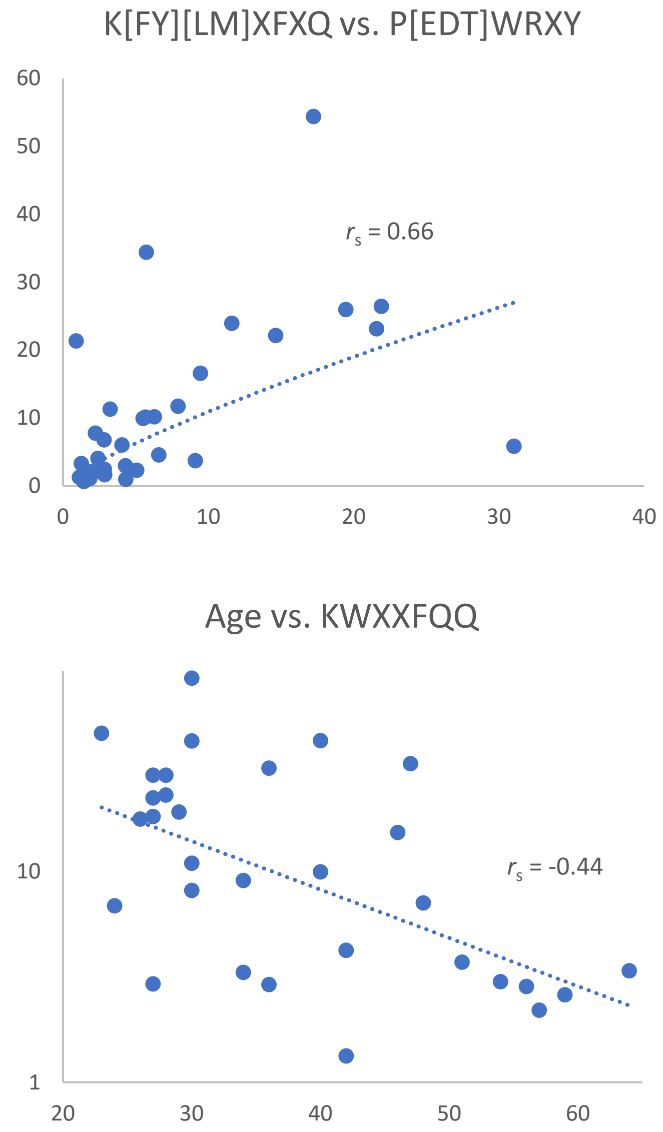

S2 Fig. Correlation of amino acid motif recognition and age of vaccinated subjects. Panel A: The fold enrichment in IgG with specificity for the amino acid motifs K[FY][LM]XFXQ vs. P[EDT]WRXY is shown. Each symbol corresponds to a different vaccinated individual. Panel B: The fold enrichment in IgG with specificity for the amino acid motif KWXXFQQ (Y-axis) is plotted against age (X-axis). Trend lines were fitted by linear regression. Spearman rank correlation (r_s) is displayed.
