# Supplementary material for: Immunogenic amino acid motifs and linear epitopes of COVID-19 mRNA vaccines
Source: PLoS One. 2021 Sep 9;16(9):e0252849. doi: 10.1371/journal.pone.0252849 (PMC8428655; doi:10.1371/journal.pone.0252849)
Supplement: S3 Fig — (PDF) [file pone.0252849.s003.pdf]

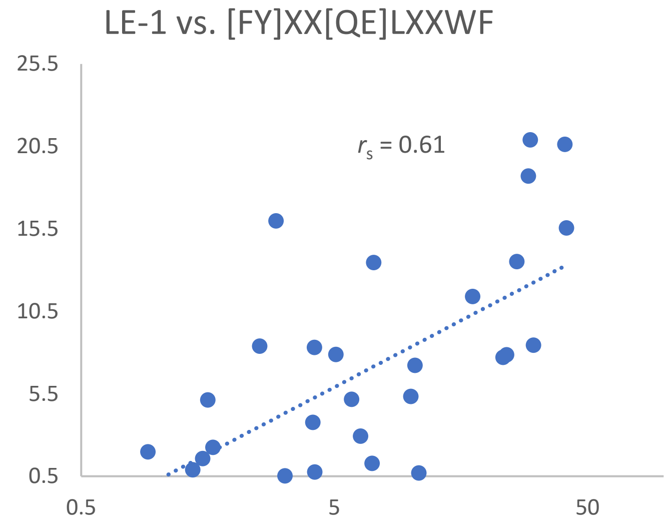

S3 Fig. Correlation between IgG recognition of amino acid motif [FY]XX[QE]LXXWF and linear epitope LE-1. The PIWAS score (Y-axis) for IgG recognition of LE-1 for individual subjects was plotted vs. the fold enrichment in IgG (X-axis) with specificity for the amino acid motif [FY]XX[QE]LXXWF in subjects that received mRNA vaccine. Each symbol corresponds to a different vaccinated individual. Trend lines were fitted by linear regression. Spearman rank correlation (r_s) is displayed.
