# Supplementary material for: Immunogenic amino acid motifs and linear epitopes of COVID-19 mRNA vaccines
Source: PLoS One. 2021 Sep 9;16(9):e0252849. doi: 10.1371/journal.pone.0252849 (PMC8428655; doi:10.1371/journal.pone.0252849)
Supplement: S4 Fig — (PDF) [file pone.0252849.s004.pdf]

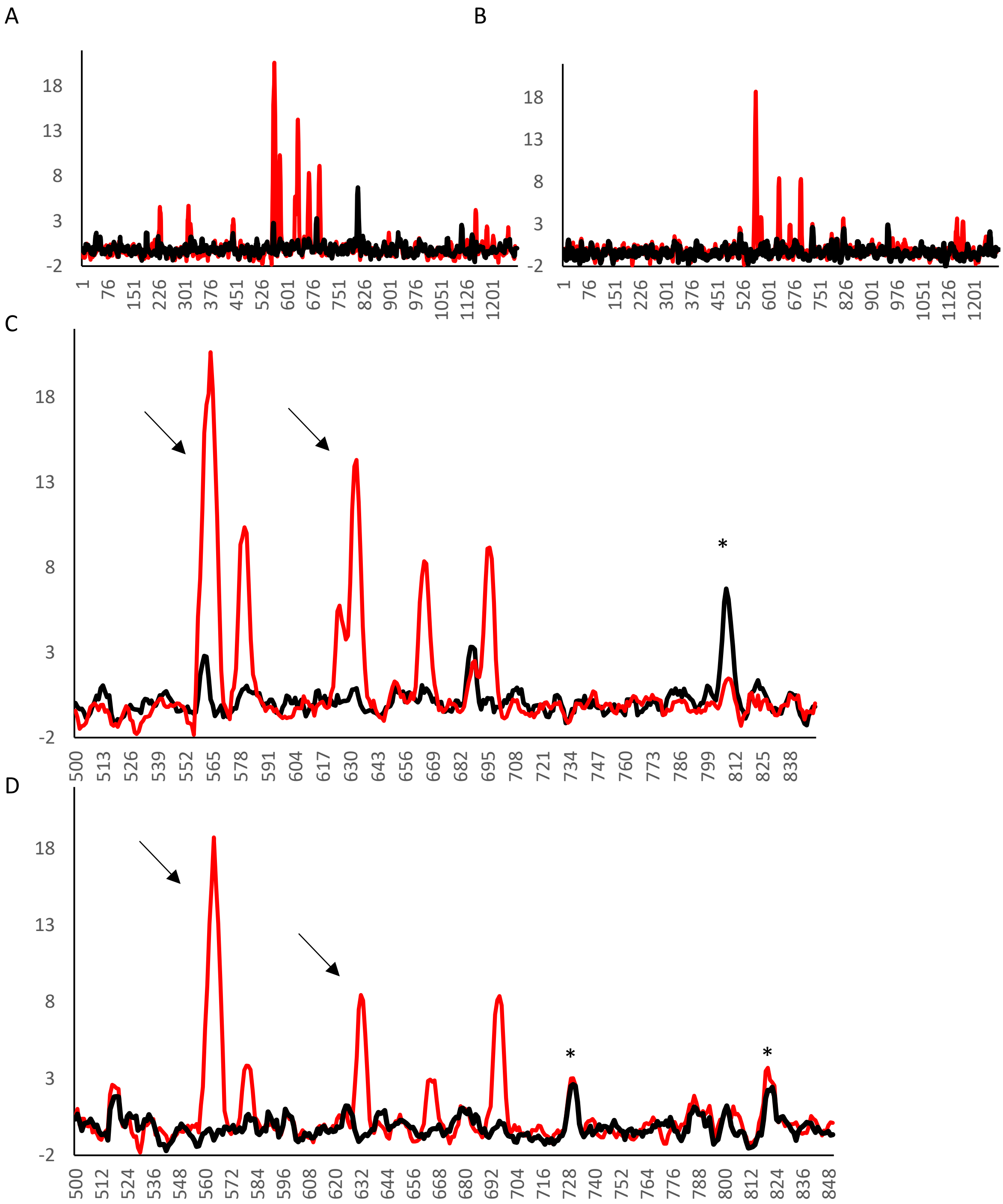

S4 Fig. Examples of epitopes that drive IgG responses in mRNA vaccinated subjects with prior COVID-19. The PIWAS scores (Y-axis) are graphed for peptides surrounding different amino acids along the SARS-CoV-2 spike protein (X-axis) that are recognized by IgG after COVID-19/pre-vaccine (black) and after COVID-19 and vaccination (red). Panels A (subject 32) and B (subject 33) show entire spike proteinand Panels C and D respectively focus on the region surrounding the S1/S2 junction. Note dominant epitope LE-1 and LE-2 (→) in both subjects and lack of substantial boost to highlighted naturally occurring epitopes (*)
