# Supplementary material for: Immunogenic amino acid motifs and linear epitopes of COVID-19 mRNA vaccines
Source: PLoS One. 2021 Sep 9;16(9):e0252849. doi: 10.1371/journal.pone.0252849 (PMC8428655; doi:10.1371/journal.pone.0252849)
Supplement: S1 Correlation matrix — (PDF) [file pone.0252849.s008.pdf]

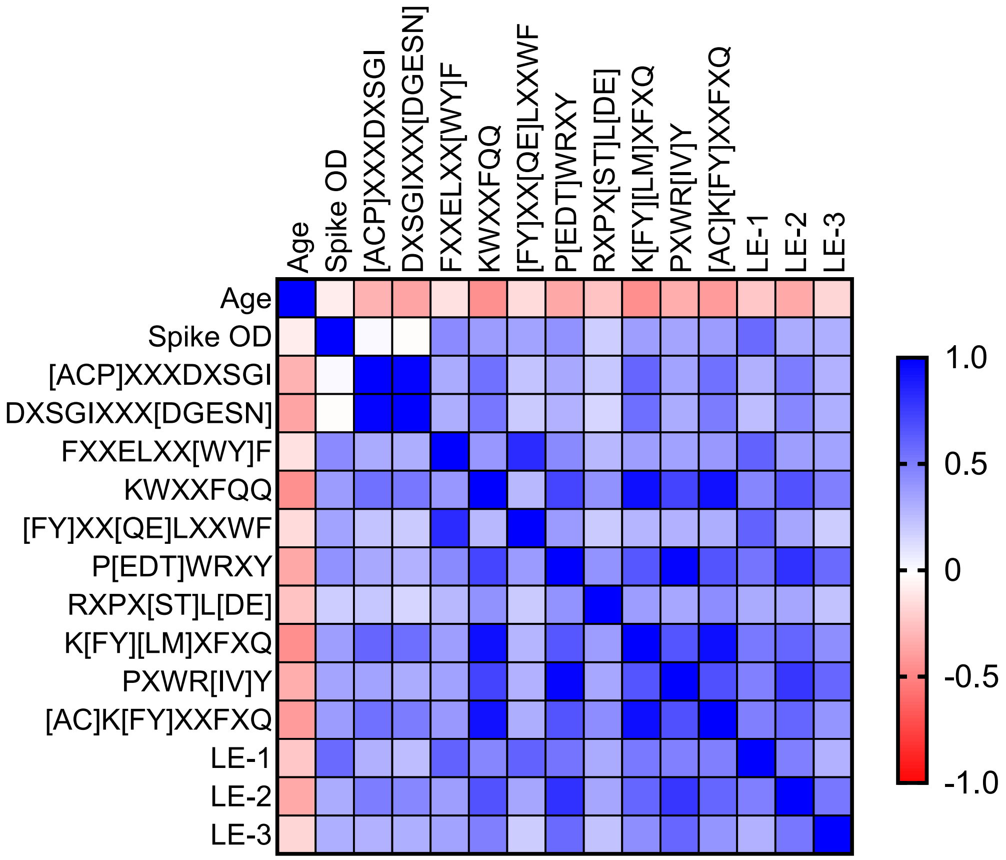

S1 Correlation Matrix. The correlation between enrichment in IgG recognition of amino acid motifs and linear epitopes. Spearman rank correlation values are displayed as a heat map by color according to key on right for different epitopes recognized by IgG from COVID-19 mRNA vaccine recipients.
