# Supplementary material for: Immunogenic amino acid motifs and linear epitopes of COVID-19 mRNA vaccines
Source: PLoS One. 2021 Sep 9;16(9):e0252849. doi: 10.1371/journal.pone.0252849 (PMC8428655; doi:10.1371/journal.pone.0252849)
Supplement: S1 List — (PDF) [file pone.0252849.s009.pdf]

S1 List. Peptides with strongest identity to SARS-CoV-2 spike mRNA vaccine epitopes identified though BLAST® analysis (https://blast.ncbi.nlm.nih.gov/Blast.cgi?PAGE=Proteins)

1) LE-1 Epitope: FLP*FQQFGRDIA*

```
>translation elongation factor Ts [Candidatus Omnitrophica bacterium]

Sequence ID: MBI1977219.1 Length: 198

Query  4   FQQFGRDIA  12

           FQQFGRDIA

Sbjct  92  FQQFGRDIA  100
```

2) LE-2 Epitope: *DQLTPTWRVY*

```
>hypothetical protein [Thermoplasmata archaeon]

Query  1   DQLTPTWRV  9

           DQLTPTWRV

Sbjct  39  DQLTPTWRV  47
```

3) LE-3 Epitope: F*KEELDKYFKN*

```
>MULTISPECIES: universal stress protein [Aquimarina]

Sequence ID: WP_074407094.1 Length: 283

Query  2    KEELDKYFKN  11

            KEELDKYFKN

Sbjct  205  KEELDKYFKN  214
```
